# A case of esophageal candidiasis in a psoriatic patient treated with ixekizumab: Should treatment be discontinued?

**DOI:** 10.1111/dth.15361

**Published:** 2022-02-14

**Authors:** Angelo Ruggiero, Matteo Megna, Vincenzo Marino, Luca Costanzo, Gabriella Fabbrocini, Sonia Sofía Ocampo‐Garza, Chiara Miano, Lucia Gallo

**Affiliations:** ^1^ Section of Dermatology, Department of Clinical Medicine and Surgery University of Naples Federico II Napoli Italy; ^2^ Servicio de Dermatología Hospital Universitario “Dr. José Eleuterio González”, Universidad Autónoma de Nuevo León San Nicolás de los Garza Mexico

Dear Editor,

Psoriasis is a chronic inflammatory skin disease with genetic background and autoimmune pathogenic traits.^1^, ^2^ Biotechnological therapy is a treatment option in moderate‐to‐severe psoriasis, with favorable benefit–risk balance and no cumulative organ‐specific toxicity, which represented one of the most important major research advantages in the management of more severe and unresponsive forms of psoriasis.^3^, ^4^, ^5^ However, biological treatments have been linked to an increased risk of infections.^4^ Interleukin (IL)‐17 is involved in mucocutaneous defense against extracellular pathogens including *Candida albicans*. Indeed, patients suffering from IL‐17 deficiency show chronic or persistent oral candidiasis from early childhood.^6^ As a result, inhibiting IL‐17 may increase susceptibility to staphylococcal infections and mucocutaneous candidiasis.^7^ Ixekizumab, a humanized‐monoclonal‐immunoglobulin‐G‐(IgG)‐4 antibody, specifically binding IL‐17A, demonstrated strong efficacy and safety profiles in treating moderate‐to‐severe psoriasis.^8^ We present the case of a 55‐year‐old man who developed esophageal‐candidiasis (EC) while being treated with ixekizumab, which, after the EC therapy, has been successfully retreated with ixekizumab. The patient referred to our outpatient clinic in April 2019 with a history of plaque‐psoriasis of 8 years. His medical history was positive for hypertension. Dermatologic examination revealed a severe and widespread form of plaque‐psoriasis (Psoriasis‐area‐and‐severity‐index [PASI]: 18; body‐surface‐area [BSA]: 35%). Treatment with standard‐dose ixekizumab led to achieving complete remission (PASI100) after 12 weeks. However, after 18 weeks, the patient started to experience growing dysphagia, first with solids and then with liquids. Esophagogastroduodenoscopy (EGD) findings resulted diagnostic for EC (Figure 1A,B). Hence, ixekizumab was suspended and candidiasis therapy started (itraconazole 200 mg per‐day for 3 weeks, then, 100 mg 7 days‐per‐month for 2 months). After 1 month, the patient reported a complete remission of dysphagia and follow‐up EGD confirmed complete EC remission after 2 months (Figure 1C,D). As regard psoriasis, after ixekizumab suspension, the patient experienced a burden of the diseases (PASI:9‐BSA:15%). Hence, ixekizumab was restarted, showing at week‐4 a huge improvement of both PASI and BSA (PASI:3‐BSA:5%). Complete remission (PASI100) was again reached at week‐8. No candidiasis or other infections were reported until the last follow‐up visit (week‐40).

**FIGURE 1:**
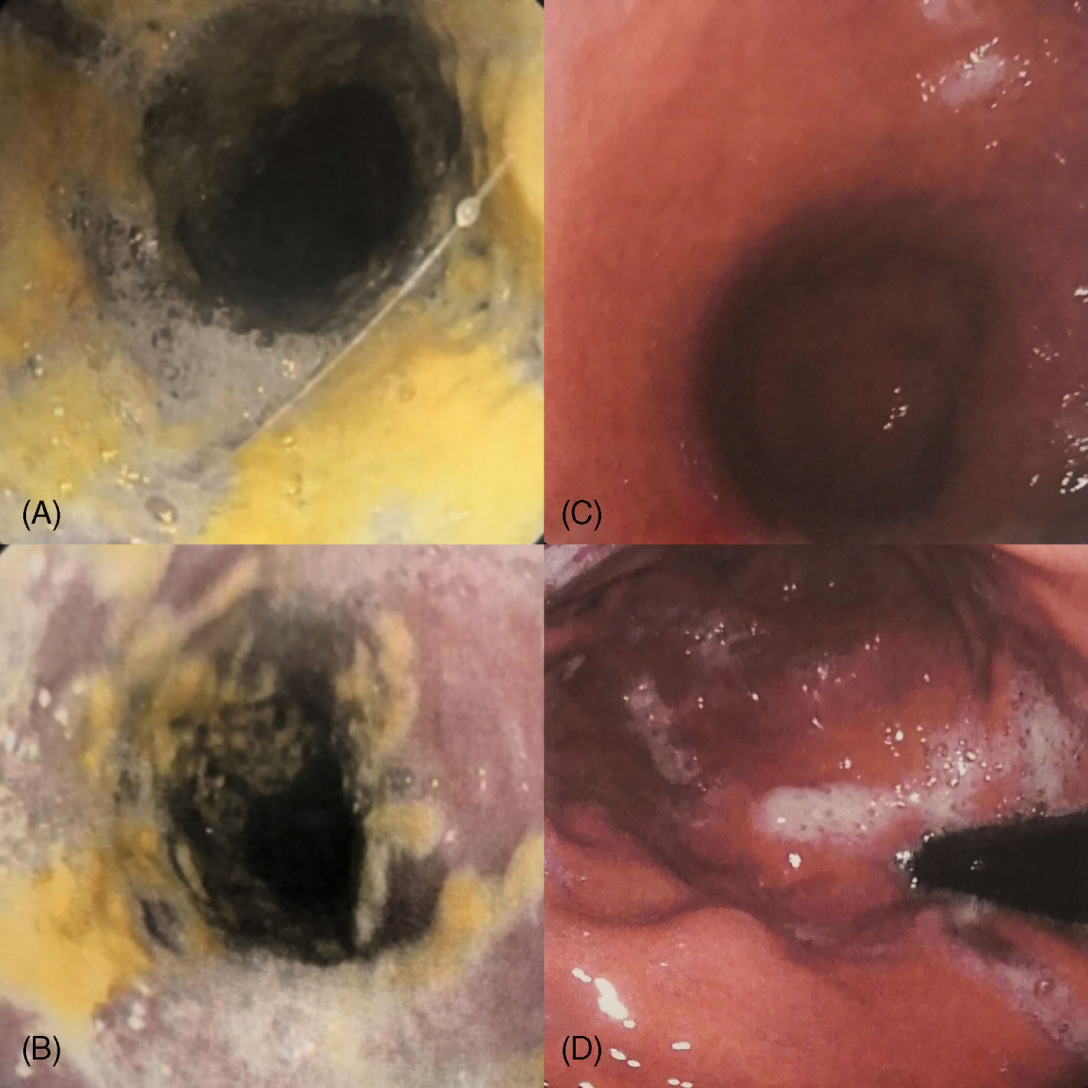
(A and B) Esophagogastroduodenoscopy (EGD) findings diagnostic for esophageal candidiasis after 18 weeks of ixekizumab and (C and D) after itraconazole 200 mg per‐day for 3 weeks, then, 100 mg 7 days per‐month for 3 months

Biologic therapies targeting proinflammatory mediators have made considerable progress in psoriasis treatment.^9^ However, these can to impair the immune system and increase infection risk, including fungal infections.^9^ Concerning IL‐17 inhibitors, many cases of *Candida Albicans* infections have been reported with secukinumab and ixekizumab.^9^ In a combined report of 2400 patients comparing ixekizumab to etanercept or placebo in moderate‐to‐severe psoriasis, there were 16 (2.1%) mild‐Candida‐infections in the ixekizumab group, not requiring treatment discontinuation.^10^ As regard EC, in a study evaluating the rate of infection during ixekizumab, based on an integrated database of seven psoriasis clinical trials comprising 4209 patients, only eight cases of EC (0.2%) across seven trials were reported, four of them had EC confirmed by EGD.^4^ Interestingly, the incidence of EGD‐confirmed EC in three retrospective studies of immunocompetent healthy individuals ranged from 0.32% to 1.17%.^4^, ^11^ Thus, the rate of EC during ixekizumab may be comparable to the general immunocompetent population. Notably, our patient, after the resolution of the infection, was retreated with ixekizumab without any EC recrudescence. Similar outcomes were reported in patients withdrawn from ixekizumab after achieving PASI75, approximately half relapsed within 5 months of withdrawal; however, most patients recaptured response within 12 weeks, and the response was maintained for up to 120 weeks of retreatment.^4^ Hence, even if ixekizumab treatment has been related to a higher risk of candidiasis infection, more studies are needed to better clarify the role of ixekizumab in the EC pathogenesis as well as the potential efficacy and safety profile in case of retreatment after previous withdrawal.

## CONFLICT OF INTEREST

G. Fabbrocini acted as a speaker or consultant for Abbvie, Amgen, Eli Lilly, Janssen, Leo‐Pharma, Almyrall, Novartis, and UCB. M. Megna acted as a speaker or consultant for Abbvie, Eli Lilly, Janssen, Leo‐Pharma, and Novartis. None of the contributing authors has any conflict of interest, including specific financial interests of relationships and affiliation relevant to the subject matter or discussed materials in the article.

## AUTHOR CONTRIBUTIONS

Angelo Ruggiero, Matteo Megna, Vincenzo Marino, and Luca Costanzo contributed to review and editing, conceptualization, writing—original draft, formal analysis (lead), and writing—review and editing (equal). Sonia Sofía Ocampo‐Garza, Lucia Gallo, Chiara Miano, Gabriella Fabbrocini contributed to conceptualization (supporting); writing—original draft (supporting), writing—review and editing (equal).

## Data Availability

Data sharing not applicable to this article as no datasets were generated or analysed during the current study.
